# Microstructural design of the stalk in the crinoid *Seirocrinus* supports its pseudoplanktonic lifestyle

**DOI:** 10.1038/s41598-025-16412-8

**Published:** 2025-08-26

**Authors:** Przemysław Gorzelak, Katarzyna Janiszewska, İzzet Hoşgör, Mariusz A. Salamon

**Affiliations:** 1 Institute of Paleobiology, Polish Academy of Sciences, Twarda 51/55, 00- 818 Warsaw, Poland; 2 Exploration Department, Turkish Petroleum Corporation (TPAO), 06530 Ankara, Turkey; 3 Faculty of Natural Sciences, Institute of Earth Sciences, University of Silesia in Katowice, Będzińska Street 60, 41-200 Sosnowiec, Poland

**Keywords:** Jurassic, Türkiye, Echinodermata, crinoids, *Seirocrinus*, microstructure, stereom, Ecology, Palaeoecology

## Abstract

During geological history, some crinoids convergently deviated from a benthic lifestyle. *Seirocrinus* is an iconic example of a crinoid that adapted to a pseudoplanktonic mode of life by living attached to drift logs. While significant effort has been devoted to exploring its functional morphology and palaeoecology, little attention has been given to the microstructural design of its stalk. Here, we describe the stereom microstructure and internal architecture of the *Seirocrinus* stalk. We demonstrate that its stereom organization closely resembles that observed in extant benthic crinoids (isocrinids): coarse, irregular labyrinthic-like stereom is found in the interpetaloid zones, while the aligned, galleried stereom is extensively developed in the petaloid zones. We show that this galleried stereom displays a distinct Voronoi-like geometrical pattern, enabling strength-to-weight optimization and minimization of calcification costs. In contrast to the stalks of extant isocrinids, however, the seirocrinid stalk exhibits certain skeletal adaptations that reduce the crinoid’s weight, such as large intercolumnar cavities. Consequently, the overall weight of these pseudoplanktonic crinoids might have been lower than previously thought, and thus they may not have significantly contributed to the sinking of the raft system, which would have allowed for multi-generational colonization of the log. These results support the hypothesis that the life of the *Seirocrinus*-bearing raft “colony” was more dependent on the wood structure itself and did not rely heavily on the seirocrinid “colonies” causing overloading.

**Supplementary Information:**

The online version contains supplementary material available at 10.1038/s41598-025-16412-8.

## Introduction

*„For sessile benthic organisms*,* becoming pelagic is an evolutionary step comparable to the acquisition of flight in land animals and likewise requires ecological stepping-stones.*^1^*” (Seilacher and Hauff*,* 2004*,* p. 14)*.

Crinoids evolved as benthic suspension feeders. However, at different times in their evolutionary history, some clades convergently evolved morphologies adapted to a pseudoplanktonic mode of life. An iconic example includes the pentacrinitid crinoid genus *Seirocrinus*, widely recorded in the Upper Triassic-Middle Jurassic strata in nearly all continents^2^.

The pseudoplanktonic mode of life of this crinoid genus has become a subject of much controversy in the past^3,4^ but nowadays it is widely accepted^5–8^. Arguments supporting a pseudoplanktonic lifestyle for *Seirocrinus* have relied on: (i) morphologic grounds (e.g., development of an extremely long (up to about 20 m) stalk displaying tapering and being more flexible distally as well as having an enlarged crown containing endotomously branching arms, which both are considered critical to the “tow-net filtration” function)^1,9–12^, (ii) taphonomic and biostratinomic evidence (e.g., wide geographic distribution and common preservation in anoxic facies, association with driftwood, preferential colonization of the regions of least resistance and free of bark, i.e., the back of the floating log)^8,12,13,14^ and (iii) quantitative analyses (spatial distribution analyses confirming preferential settlement at the terminal log end, and diffusion modelling suggesting prolonged soaking durations of wood logs)^8^.

Predictably, the unusual morphology of *Seirocrinus* has garnered much attention, but relatively little study was devoted to understanding microstructural design and internal architecture of their stalk. Seilacher et al. ^9^ and Seilacher and Hauff^1^ have argued that the nodals of these crinoids were formed in the more stiff proximalmost part of the stalk which was protected by aboral extensions of the basals and dense set of cirri, and that the extreme extension of the stalk was possible thanks to unlimited formation of internodals between two biconcave nodals. More recently, Hagdorn^14^ briefly mentioned that *Seirocrinus* developed a lightweight construction to reduce the load of the driftwood by “lensoid intercolumnar spaces and long intercolumnar collagen fibers” and “a distally tapering column” (Hagdorn^6^; p. 239). While these studies indicate that *Seirocrinus* might have developed some skeletal adaptations to reduce weight, neither documents in detail any microarchitectural features of their stalks. Here, we describe the stereom microstructure and internal morphology of the well-preserved stalk of *Seirocrinus* and discuss their possible functional interpretations.

## Results

**Fig. 1:**
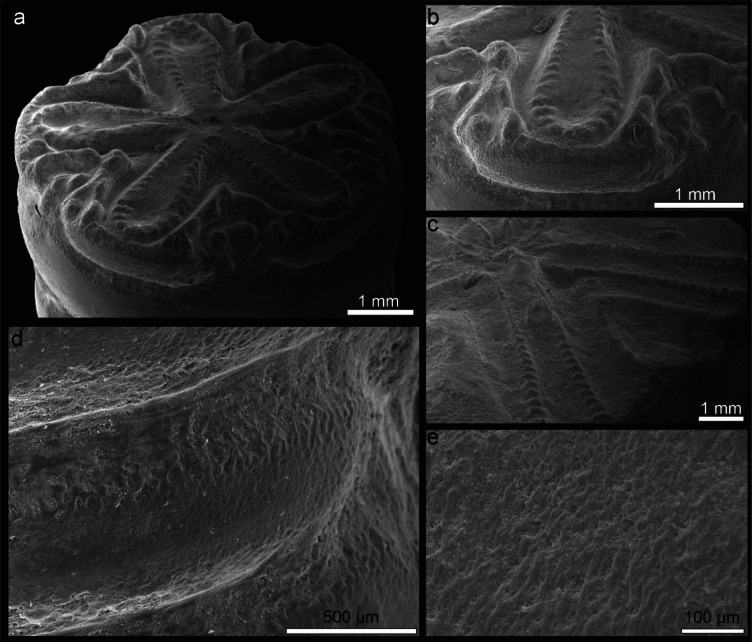
General morphology of the *Seirocrinus* columnals. (**a**) Outline of morphological facet. (**b**,** c**) Enlargements of petaliod and interpetaloid areas, respectively. (**d**) Columnal latera. (**e**) Enlargement of stereom microstructure of the columnal latera. GIUS 8–3689/1.

**Fig. 2:**
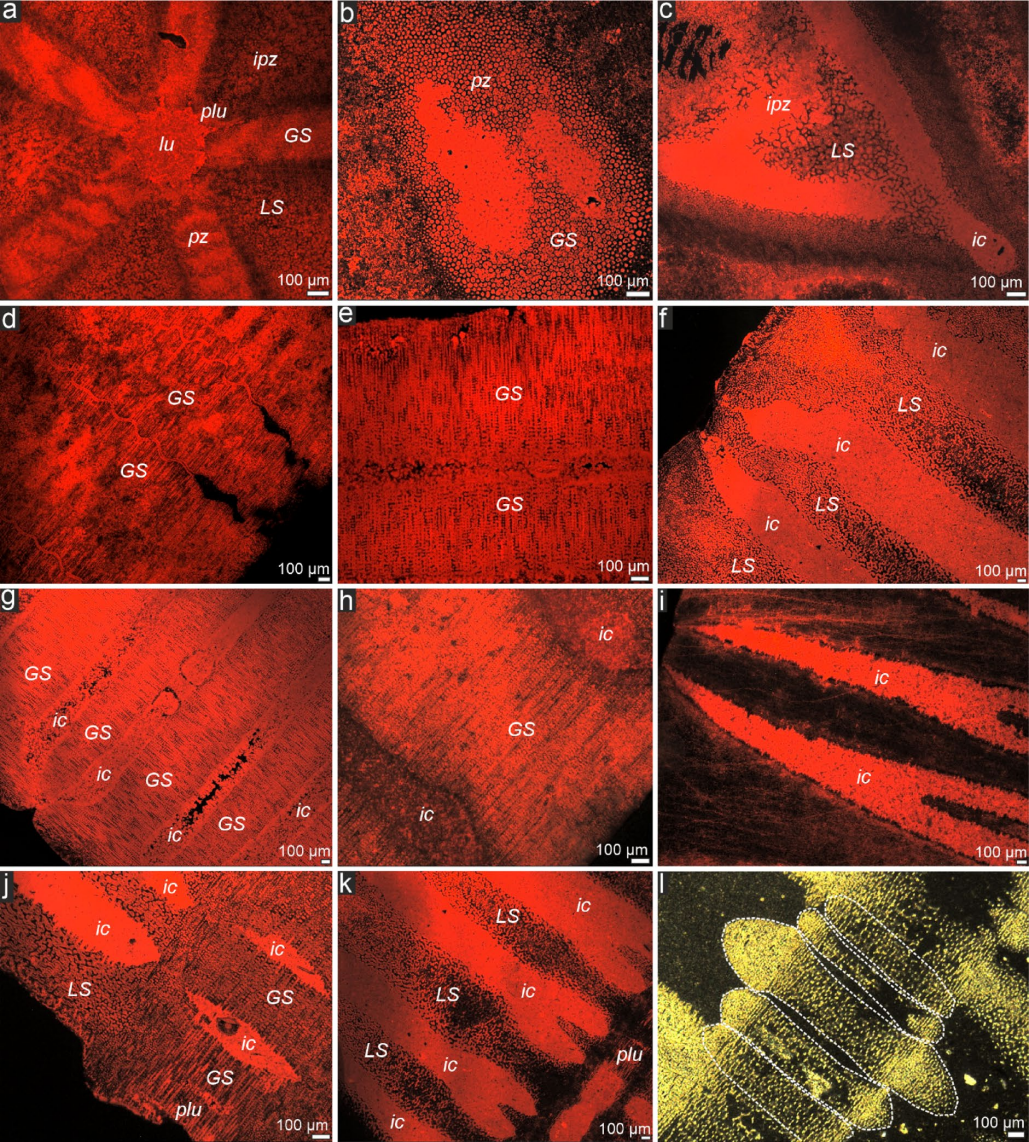
Stereom organization of the stalk of *Seirocrinus* (CL [a-k] and optical [l] micrographs). (**a**) Transverse section through the central part of the columnal (lumen, perilumen and parts of areola and interpetaloid zones). (**b**,** c**) Enlargement of petaloid and interpetaloid zones, respectively (transverse planes). (**d**,** e**) Longitudinal sections through/near crenularium of a pluricolumnal. (**f**) Longitudinal sections through interpetaloid zone of a pluricolumnal. (**g**,** h**) Longitudinal sections through inner parts of petaloids of a pluricolumnal. (**i**–**k**) Longitudinal sections through pluricolumnal showing large intercolumnal cavities, note transition between galleried stereom of perilumen and coarse perforate labirynthic stereom of interpetaloid zone (**j**). (**l**) Longitudinal section through a pluricolumnal showing five immature thin columnals (white dotted lines) in large cavity between two fully grown concave columnals. GS - galleried stereom, LS - coarse labyrinthic stereom, lu - lumen, plu - perilumen, pz - petaloid zone, ipz - interpetaloid zone, ic - intercolumnal cavities. GIUS 8–3689/Tur10.

**Fig. 3:**
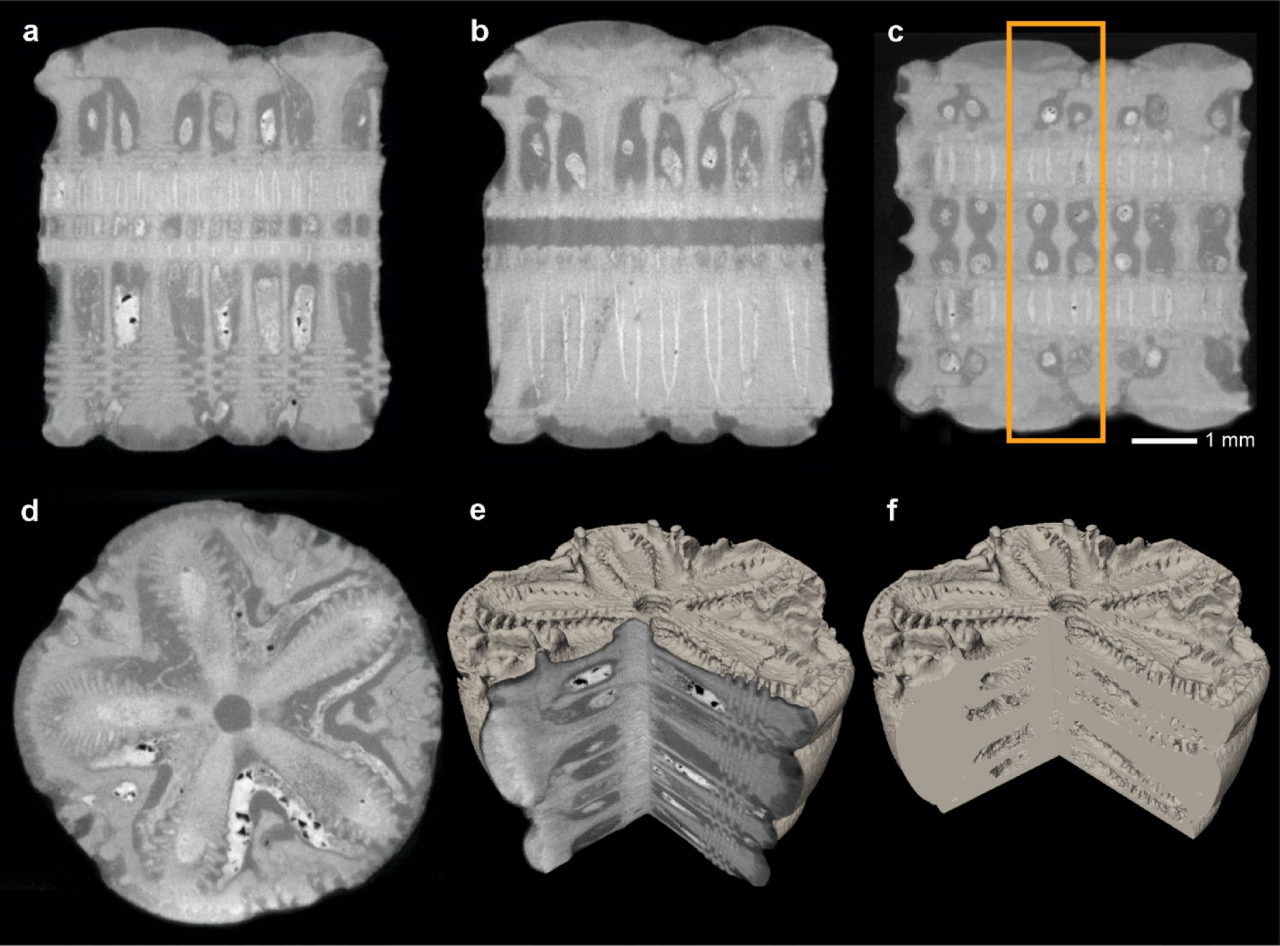
Cross-sections through a fragment of *Seirocrinus* stem obtained by microtomography illustrating intercolumnal hollow structures. (**a**–**c**) Longitudinal sections. (**d**) Transverse section. (**e**,** f**) Visualisations of investigated pluricolumnal showing the distribution of internal cavities after virtual removal of diagenetic fillings. Orange frame in c shows the area taken into account for calculating the percentage of internal void volume. GIUS– 3689/9, 2.

Examined median columnals of *Seirocrinus* reveal some hollow structures and grooves (so-called rugose pattern) in the radial sectors of articular facets (Fig. 1a–c). In transverse sections, strongly biconcave columnals can be noted (Supplementary videos 1–2; see also Figs. 2d–l and 3); between them - within enlarged cavities, up to about 5 newely forming thin immature internodals can be observed (Fig. 2l). The volume of intercolumnal cavities constitutes ca. 20% of the total volume of the pluricolumnal fragment analysed by X-ray microtomography (micro-CT).

The general stereom organization in seirocrinid columnals is very similar to that observed in some Recent isocrinids^15,16^. More specifically, coarse (trabecular thickness: 9–25 μm), irregular (pore diameters: 3–16 μm) labyrinthic-like stereom is observed in the interpetaloid zones and in other abcentral parts of the articulum and in the columnal latera (Figs. 1d and e and 2c, f, j and k), whereas the galleried stereom is well developed in the perilumen and in the petaloid areola (including crenulae) (Fig. 2a, b, d, e). The galleried stereom is composed of straight, parallel trabecular rods oriented perpendicular to the columnal facets (Fig. 2d, e, g, h).

**Fig. 4:**
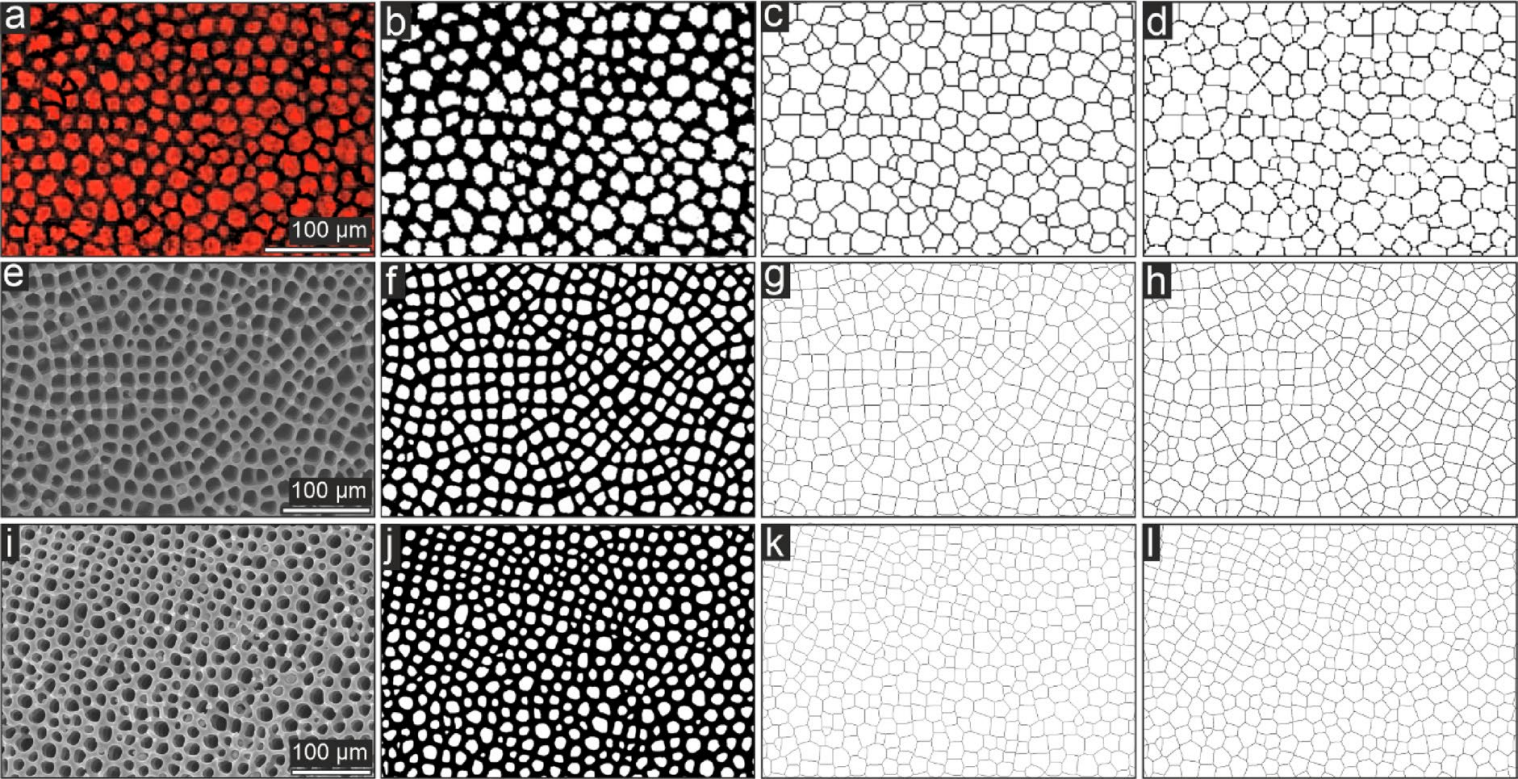
Voronoi divergence analysis of petaloid stereom in *Seirocrinus* (**a**–**d**), *Hypalocrinus* (**e**–**h**) and *Metacrinus* (**i**–**l**). CL or SEM micrographs (**a**,** e**,** i**), micrographs binarizations (**b**,** f**,** j**), skeletonizations of the binarized images (**c**,** g**,** k**), computations of the Voronoi model (**d**,** h**,** l**).

In transverse planes, the galleried stereom of *Seirocrinus* and the Recent benthic isocrinids resembles the so-called Voronoi diagram: it is composed of the polygonal cell shapes (Fig. 4) dominated by hexagons, followed by heptagons and pentagons, with minor contribution of quadrilaterals, octagons and triangles. The basic stereom parameters of petaloids are given in Table 1.

**Table 1:** Summary of trabecular and pore analysis results. *divergence estimation between the trabecular system and the generated Voronoi model (% Absolute Error Count); note that the higher divergence in *Seirocrinus* is affected by artefacts related to diagenetic processes (secondary cement infillings of the stereom pores makes their separation (using a thresholding) difficult).

	*Seirocrinus*	*Metacrinus*	*Hypalocrinus*
Porosity (%)	51.03	49.56	51.14
Mean pore area (µm^2^)	92.71	135.7	107.66
Circularity	0.87	0.89	0.85
Mean branch length (µm)	9.8	11.8	10.4
Triple points (%)	96.4	96.97	95.4
Triangles (%)	0	0	0.7
Quadrilaterals (%)	5.23	4.47	9.15
Pentagons (%)	16.99	21.79	15.49
Hexagons (%)	54.9	46.09	36.27
Heptagons (%)	19.61	23.18	28.87
Octagons (%)	3.27	4.47	9.51
Voronoi (error %)*	15	5.27	5.47

## Discussion

Seirocrinids evolved a number of morphological features adapted to a pseudoplanktonic mode of life^1,6^. Most importantly, they significantly remodelled their stalk. In particular, to increase efficiency of tow-net filtration, pseudoplanktonic crinoids needed to develop much longer, faster growing and distally more flexible stalks than their benthic ancestors to occupy water with different velocities and nutrient concentrations at different depths within a limited drifting time^1,17^. This was achived by simultaneous formation of new thin columnals between two biconcave columnals along the stem, which gradually tapered towards the distal end.

The stem of pseudoplanktonic crinoids must have been also resistant to tangling and breakage. To accommodate these functional requirements, the *Seirocrinus* stem was stitched by long through-going ligaments, which is evidenced by the presence of galleried strereom consisting of long trabecular passageways deeply extending into the columnal interiors and passing through many columnals within the stalk (Fig. 2d, g). This stereom configuration is diagnostic of long through-going ligaments^18^. However, this stereom arrangement has already been observed in early fossil crinoids, suggesting it is a conservative trait^19^. Notably, in contrast to Recent benthic isocrinids, *Seirocrinus* lacks the so-called synostosial stereom that is associated with short ligament fibers specifically designed for autotomy^20^. Thus, it seems that tensile and torsional strength, as well as flexibility in *Seirocrinus* could have been achieved by distally tapering columns, devoid of autotomy planes, that were filled with long through-going collagenous tissues.

Intriguingly, we found that the stereom in petals of *Seirocrinus* and Recent benthic isocrinids is arranged into polygonal cells, resembling the so-called Voronoi pattern. In a Voronoi diagram, the plane is divided up into polygons (cells), one around each point, in such a way that each region from the area surrounding a given point is closer to it than to any other point^21^. This configuration offers the most efficient way of packing shapes in a plane. It is thus not surprising that a Voronoi pattern is commonly observed in nature (e.g., in cells, mammalian coat patterns etc.) and architecture. In the case of echinoderms, Perricone et al.^22^ have recently recognized a very similar Voronoi-like stereom organization in the tubercle boss of a Recent echinoid species, *Paracentrotus lividus*, and interpreted it as an evolutionary solution for enhancing the structural strength and minimizing the metabolic costs of calcification. Such “optimization” was likely particularly advantageous for *Seirocrinus*, given the necessity of producing a long, rapidly growing, and mechanically stiff stalk. Notwithstanding, our data indicate that a similarly “optimized” stereom organization is also present in extant benthic crinoids, suggesting that this feature is more widespread among echinoderms than previously assumed.

One of the main arguments once put forward against the hypothesis of a pseudoplanktonic mode of life of seirocrinids concerns larval settlement. After their swimming stage, it seems unlikely that larvae could reliably locate the rare substrate of driftwood in the open ocean. However, this may have been more feasible in the Early Jurassic, before the widespread proliferation of wood-boring bivalves. Reduced biotic degradation would have allowed driftwood to remain buoyant longer, increasing opportunities for larval attachment and colonization. Feeding efficiency has been also questioned in pseudoplanktonic crinoids, as weak currents around driftwood may not suport effective suspension feeding especially in juvenile forms with shorter stalks. However, Hess^5^ suggested that pseudoplanktonic crinoids likely employed diverse strategies, including tow-net filtration, feeding on phytoplankton near driftwood, and interception of vertically migrating zooplankton during diel cycles. Another challenge to the pseudoplanktonic model concerns the longevity of the raft system: how long would the log have to remain on the ocean surface for the seirocrinids to grow 20-metre-long stalks? Data presented herein suggest that seirocrinids were rather lightweight organisms which acquired some structural adaptations that might have increased buoyancy. Consequently, they would not contribute much to the sinking of the raft system. The internal hollow morphology of the *Seirocrinus* stalks, visualized in detail herein for the first time, has been found to account for approximately 20% of the volume of the stem (in addition note that the stereom itself is also porous). These data strengthen recently published results of simulations^8^ suggesting that the life of the *Seirocrinus* “colony” might have been predominantly dependent on the wood structure itself. In particular, Hunter et al.^8^ demonstrated that some of the largest Holzmaden logs (ca. 10 m in length) might have supported population of crinoids for up to about 20 years. They also showed that dense communities of crinoids (and bivalves) growing on the logs, even at their climax state, would still contribute at most 50% of the total weight of the raft system. It is noteworthy that the aforementioned authors calculated mass increments of *Seirocrinus* using the specific gravity of calcite at 2.7 g/cm^3^ (see their supplementary materials, p. 15); however, the stereom density of Recent crinoids is much lower (1.11–1.74 g/cm^3^^23^). It seems likely that *Seirocrinus* might have lowered their density even further by developing internally hollow structures. Therefore, it is likely that the *Seirocrinus* “colonies” contributed to a limited extent to the lifespan of the raft system, which enabled multi-generation colonization of the logs by *Seirocrinus* (cf., a four-generation “colony” described by Matzke and Maisch^7^). Instead, the collapse of the log raft was primarily due to the progressive infiltration of water and the subsequent decay of the wood over time.

## Concluding remarks

In their long evolutionary history, some crinoid clades have occasionally deviated from their prototypical Bauplan well adapted to benthic mode of feeding, and evolved into “pelagic” ecological niches. Among such crinoids are *Seirocrinus*. These crinoids utilized a pseudoplanktonic tow-net mode of feeding, which was facilitated by the development of an enlarged and dense crown, as well as a unique stalk, which, despite its similarity to modern benthic crinoids, displays a distinct combination of morphological features. More specifically, simultaneous formation of new columnals all along the stalk increased its growth rate, allowing it to reach lengths of up to 20 m. This enabled the filtration fan and the distal, driftwood end of these crinoids to occupy water with varying flow regimes and velocity gradients. The development of long, continuous intercolumnal ligaments and the loss of autotomy planes made the stem both flexible and resistant to tangling and breakage. Furthermore, the large intercolumnal cavities may have increased the crinoid’s overall buoyancy, which was crucial for extending the soaking durations of the log raft system, allowing multi-generational colonization of the log.

## Materials and methods

Well preserved stalk fragments (from the median part of the stalk) of *Seirocrinus* stored at the Faculty of Earth Sciences, University of Silesia in Katowice, Poland (acronymed under catalogue numbers: GIUS 8–3689/1–9, GIUS 8–3689/Tur10), and at the Centre for Nature Education, Jagiellonian University in Kraków, Poland (acronymed under catalogue number: CEP-DG–775) were investigated. The fossils acronymed under catalogue GIUS 8–3689/Tur10 were collected by one of us (İH) from the lower part of the Bayırköy Formation (Late Sinemurian-Pliensbachian^24,25^) in the northeastern part of the Ankara, around the village of Kösrelik, Türkiye. Other materials include two pluricolumnals (GIUS 8–3689/1, 2) collected from Pliensbachian of Algeria (The Ain Ouarka Formation) and several isolated stalk fragments (CEP-DG–775) derived from the Lower Jurassic (Toarcian), around Holzmaden, Germany, both recently described by Kajdas et al.^26^ and Salamon et al.^27,28^.

Observations of general morphology and stereom microstructure of the columnals (Fig. 1) were conducted using a Philips XL–20 scanning electron microscope (SEM) at the Institute of Paleobiology of the Polish Academy of Sciences in Warsaw, Poland (accelerating voltage ca. = 25 kV, working distance ca. = 34 mm). Additionally, 20 variously oriented thin sections of *Seirocrinus* stalks polished down to about 25–50 μm were coated with carbon and examined with a cathodoluminescence (CL) (Fig. 2), which was proved as a useful method for investigating the skeletal microstructures of fossil echinoderms^29^. The latter observations were performed with the aid of a Lumic HC5–LM microscope equipped with a hot cathode at the Institute of Paleobiology of the Polish Academy of Sciences in Warsaw (an electron energy = 14 keV, a beam current = 0.03–0.1 mA).

In order to geometrically describe the stereom pattern of petaloids, we selected one CL image (the least “blurred”), showing a microregion with the strongest contrast between cement infill and the stereom (Fig. 4). For comparison purpose, we also acquired two images of columnal petals of two species of Recent stalked isocrinine (*Metacrinus* and *Hypalocrinus*; for details about materials and sampling sites see^30^). Following the approach outlined by Perricone et al.^22^ we then quantified basic geometrical stereom parameters for both *Seirocrinus* and Recent isocrinids using ImageJ Fiji software (v. 1.52e): porosity (amount of pore area in the skeleton compared to the skeletal area), mean pore area (calibrated square units µm^2^), circularity (shape descriptor for which a value of 1 indicates a perfect circle and values decreasing toward 0 indicate increasingly elongated shapes), mean branch length, number of junctions per each node, number of neighbour seeds, divergence (in %) between skeletal pattern and a Voronoi model (computed using the Delaunay Voronoi algorithm).

Micro-CT data were collected with Zeiss XRadia MicroXCT-200 system equipped with a 90 kV/8 W tungsten X-ray source in the Laboratory of Microtomography, Institute of Paleobiology, Polish Academy of Sciences, Warsaw, Poland. The scan was performed using the following parameters: voltage: 70 kV; power: 6 W; exposure time: 4 s; voxel size: 11.20 μm. Radial projections were reconstructed with XMReconstructor software. The virtual sections, 3D visualisations and internal void volume measurements were prepared with Avizo Fire software.

## Supplementary Information

Below is the link to the electronic supplementary material.

Supplementary Material 1

Supplementary Material 2

Supplementary Material 3

## Data Availability

The data presented in this manuscript, including those derived from tomography, are sufficient for interpretation and further morphological analyses. The original tomographic datasets are available from the corresponding author upon request (Przemysław Gorzelak (pgorzelak@twarda.pan.pl)).
