# Supplementary material for: Microstructural design of the stalk in the crinoid Seirocrinus supports its pseudoplanktonic lifestyle
Source: Sci Rep. 2025 Aug 26;15:31418. doi: 10.1038/s41598-025-16412-8 (PMC12381209; doi:10.1038/s41598-025-16412-8)
Supplement: Supplementary file 1 — Supplementary Material 1 [file 41598_2025_16412_MOESM1_ESM.pdf]

Supplementary Information for the article

Microstructural design of the stalk in the crinoid *Seirocrinus* supports its pseudoplanktonic lifestyle

Przemysław Gorzelak[1*], Katarzyna Janiszewska[1], İzzet Hoşgor[2], and Mariusz A. Salamon[3*]

[1*]Institute of Paleobiology, Polish Academy of Sciences, Twarda 51/55, 00-818 Warsaw, Poland

[2]Turkish Petroleum Corporation (TPAO), Exploration Department, 06530, Ankara, Türkiye

[3]University of Silesia in Katowice, Faculty of Natural Sciences, Institute of Earth Sciences, Będzińska Street 60, 41-200 Sosnowiec, Poland

Corresponding Authors:
pgorzelak@twarda.pan.pl and paleo.crinoids@poczta.fm

Supplementary video 1. 3D animation of *Seirocrinus* pluricolumnal (transverse section) showing internal hollow structures (marked in blue).

Supplementary video 2. 3D animation of *Seirocrinus* pluricolumnal (longitudinal section) showing internal hollow structures (marked in blue).
